# Association between white matter T1w/T2w ratio and cognitive function in FTD genetic mutation carriers

**DOI:** 10.1002/alz70856_105626

**Published:** 2026-01-07

**Authors:** Hyunwoo Lee, Ian R MacKenzie, Mirza Faisal Beg, Karteek Popuri, Dana Wittenberg, Winston Huang, Ging‐Yuek Robin Hsiung

**Affiliations:** ^1^ Division of Neurology, Department of Medicine, University of British Columbia, Vancouver, BC, Canada; ^2^ University of British Columbia, Vancouver, BC, Canada; ^3^ Simon Fraser University, Burnaby, BC, Canada; ^4^ Memorial University Of Newfoundland, St. John's, NF, Canada

## Abstract

**Background:**

Frontotemporal dementia (FTD) presents with heterogeneous, progressive deficits in behavior, language, and cognition. These changes have been associated with white matter (WM) alterations on MRI, including white matter signal abnormalities and diffusion‐based metrics. Recent pathological findings suggest that white matter changes in FTD, particularly in individuals with mutations in the progranulin gene (*GRN*), may be partly attributable to myelin deficits. The ratio of T1‐weighed and T2‐weighted images on MRI (T1w/T2w) has been demonstrated as an indicator of myelin content in the brain. We hypothesized that *GRN* mutation carriers would exhibit a reduced T1w/T2w ratio compared to those with mutations in the chromosome 9 open reading frame 72 *(C9orf7*2) or noncarriers. Additionally, we hypothesized a correlation between the T1w/T2w ratio and FTD‐related cognitive functions.

**Method:**

*GRN*, *C9orf72*, and noncarrier family controls (*N* = 80) were recruited through the University of British Columbia Familial FTD Study. Neuropsychological domains, including attention, language, visuospatial skills, working memory, verbal memory, and non‐verbal memory, were examined using neuropsychological test batteries. All cognitive domain scores were transformed into z‐scores. For each participant, T1w and T2w MRI were acquired using a 1.5T scanner. T1w and T2w images were spatially coregistered, followed by intensity standardization. Average T1w/T2w was calculated within the WM region‐of‐interest defined by the JHU WM Tractography Atlas. This was a cross‐sectional analysis using baseline images. General linear models were used to: 1) Conduct a group comparison between genetic variants; and 2) Find an association between T1w/T2w and the neuropsychological domains. Both models were adjusted for age, sex, white matter signal abnormalities, and symptomatic status.

**Result:**

T1w/T2w was significantly lower in *GRN* carriers, especially in the frontal lobar WM. There was no significant difference between *C9orf72* and noncarriers. Lower T1w/T2w in the frontal lobe correlated with poorer working memory, language, and visuospatial scores.

**Conclusion:**

WM changes observed in *GRN* carriers may be associated with deficits in the maintenance of cerebral myelin. Furthermore, the association between cognitive changes and reduced T1w/T2w levels, particularly in the frontal lobar regions, suggests that further studies are warranted to understand the role of *GRN* in myelin health and the manifestation of FTD‐related symptoms.

